# The Influence of Dapagliflozin on Foot Microcirculation in Patients with Type 2 Diabetes with and without Peripheral Arterial Disease—A Pilot Study

**DOI:** 10.3390/ph17091127

**Published:** 2024-08-26

**Authors:** Božena Bradarić, Tomislav Bulum, Neva Brkljačić, Željko Mihaljević, Miroslav Benić, Božo Bradarić Lisić

**Affiliations:** 1 Vuk Vrhovac University Clinic for Diabetes, Endocrinology and Metabolic Diseases, Merkur University Hospital, 10000 Zagreb, Croatia; 2 School of Medicine, University of Zagreb, 10000 Zagreb, Croatia; 3 Croatian Veterinary Institute, 10000 Zagreb, Croatia; 4 Professional Study Program in Physiotherapy, University of Applied Health Sciences, 10000 Zagreb, Croatia

**Keywords:** microcirculation, transcutaneous oxygen pressure (TcPO2), peripheral arterial disease (PAD) diabetes mellitus, SGLT2–inhibitors

## Abstract

The results of large cardiovascular studies indicate that SGLT-2 inhibitors may increase the risk of leg amputations. This study aims to investigate whether dapagliflozin therapy affects peripheral vascular oxygenation, i.e., microcirculation in the foot, as measured by transcutaneous oxygen pressure (TcPO2) in patients with type 2 diabetes (T2DM) and peripheral arterial disease (PAD) compared to patients without PAD. The patients with PAD were randomized into two groups. In the first 35 patients with PAD, dapagliflozin was added to the therapy; in the other 26 patients with PAD, other antidiabetic drugs were added to the therapy. Dapagliflozin was added to the therapy in all patients without PAD. TcPO2 measurement, Ankle Brachial Index (ABI), anthropometric measurements, and laboratory tests were performed. After a follow-up period of 119.35 days, there was no statistically significant difference in the reduction of mean TcPO2 values between the group with T2DM with PAD treated with dapagliflozin and the group with T2DM with PAD treated with other antidiabetic drugs (3.88 mm Hg, SD = 15.13 vs. 1.48 mm Hg, SD = 11.55, *p* = 0.106). Patients with control TcPO2 findings suggestive of hypoxia (TcPO2 < 40 mm Hg) who were treated with dapagliflozin had a clinically significant decrease in mean TcPO2 of 10 mm Hg or more (15.8 mm Hg and 12.90 mm Hg). However, the aforementioned decrease in TcPO2 was not statistically significantly different from the decrease in TcPO2 in the group with PAD treated with other diabetic medications (*p* = 0.226, *p* = 0.094). Based on the available data, dapagliflozin appears to affect tissue oxygenation in T2DM with PAD. However, studies with a larger number of patients and a longer follow-up period are needed to determine the extent and significance of this effect.

## 1. Introduction

Patients with diabetes have a two to four times higher risk of developing peripheral arterial disease (PAD) than people without diabetes [1,2,3]. About 15–20% of patients with PAD and diabetes end up with limb amputation [4,5,6,7]. Non-traumatic lower limb amputations are 15 times more common in patients with diabetes than in patients without diabetes [4,5,6,7]. The factors that increase the risk of amputation are complex and include not only macrovascular disease (PAD) but also diabetic neuropathy and microvascular foot disease, which are also the main etiopathogenetic factors in the development of the diabetic foot. Diabetic sensorimotor neuropathy leads to atrophy and dysfunction of the small muscles of the foot, resulting in deformities and changes in pressure distribution in the foot. Together with reduced touch sensation and proprioception, this leads to reduced capillary blood flow to the foot and increased ischemia in areas of increased pressure, as well as an increased risk of trauma. Due to diabetic neuropathy, patients with PAD often do not have typical claudication symptoms, which further increases the risk of developing wounds. Microvascular foot disease results from hyperglycemia-induced damage to the capillary endothelium, leading to disruption of endothelial permeability and induction of a proinflammatory state, along with disruption of nutrient and oxygen supply to the tissue. An imbalance between pro- and anti-neovascularization responses occurs, resulting in reduced formation of new blood vessels in the skin, decreased repair and regenerative capacity, and induction of a procoagulant state leading to microthrombosis and subsequent tissue ischemia and damage [2,8,9]. Transcutaneous tissue oximetry (TcPO2) is a non-invasive method for measuring oxygen pressure on the skin surface to assess peripheral vascular oxygenation and microcirculation. Values below 40 mm Hg are considered pathologically low or hypoxic, which can slow or reduce wound healing, while values above 40 mm Hg are considered normal [10,11,12,13]. PAD increases the risk of cardiovascular disease and death, and these risks are even higher when diabetes is associated with PAD [14].

SGLT-2 inhibitors are now considered one of the essential antidiabetic drugs in patients with type 2 diabetes (T2DM). In clinical trials, SGLT2 inhibitors reduce the number of hospitalizations due to heart failure and slow the progression of kidney complications in patients with T2DM. According to the joint ADA/EASD guidelines, these drugs are recommended for patients with T2DM and established atherosclerotic cardiovascular disease and/or heart failure and/or chronic kidney disease and for patients at increased risk for these conditions [15,16,17,18,19,20,21,22]. However, in the CANVAS study (Canagliflozin Cardiovascular Assessment Study), patients taking SGLT-2 inhibitor canagliflozin had a twofold higher risk of amputations compared to placebo [17]. In the DECLARE-TIMI 58 study (Dapagliflozin and Cardiac, Kidney, and Limb Outcomes in Patients With and Without Peripheral Artery Disease in DECLARE-TIMI 58), a non-significantly higher number of amputations was observed in the group of patients with PAD treated with dapagliflozin compared to placebo [18].

Nowadays, diabetes is a leading cause of non-traumatic lower-extremity amputation. Since results from large cardiovascular trials indicated that SGLT-2 inhibitors might increase the risk of leg amputations, this study aims to determine whether dapagliflozin therapy affects peripheral vascular oxygenation, as measured by transcutaneous tissue oximetry, in patients with type 2 diabetes and PAD compared to patients without PAD.

## 2. Results

At baseline, we found significant differences between the groups in BMI values (*p* = 0.012), prevalence of hyperlipidemia (*p* = 0.015), prevalence of diabetic polyneuropathy (*p* = 0.001), frequency of smoking (*p* = 0.009), history of ischemic heart disease (*p* = 0.006), myocardial infarction (*p* = 0.004) and cerebrovascular disease (*p* = 0.001), and in the proportion of patients taking antiaggregation and/or anticoagulation therapy (*p* = 0.001) and lipid-lowering therapy (*p* = 0.048).

At baseline, the group of patients with PAD treated with dapagliflozin had a significantly higher BMI (28.999 vs. 26.582 kg/m^2^, *p* = 0.0075), a significantly higher prevalence of hyperlipidemia (33 vs. 17 patients, *p* = 0.006), and a larger proportion taking lipid-lowering therapy (27 vs. 13 patients, *p* = 0.027), as well as a significantly higher frequency of previous amputations (9 vs. 1 patient, *p* = 0.034), compared to the group with PAD treated with other antidiabetic drugs. The average follow-up period for all groups of patients was 119.35 days. The intergroup comparisons of all patients with and without PAD at the beginning of the follow-up are presented in Table 1 and Table 2.

Significantly higher mean BMI values at baseline were observed in patients without PAD treated with dapagliflozin compared to patients with PAD treated with other antidiabetic drugs (30.387 kg/m^2^ vs. 26.571 kg/m^2^, *p* = 0.002) and between patients with PAD treated with dapagliflozin and patients with PAD treated with other antidiabetic drugs (28.999 kg/m^2^ vs. 26.571 kg/m^2^, *p* = 0.0075). During follow-up, there was a slight decrease in BMI values in the groups without PAD and with PAD treated with dapagliflozin and a minimal increase in BMI in patients with PAD treated with other antidiabetic drugs, but the differences between the previously mentioned groups remained statistically significant (*p* = 0.010, *p* = 0.029).

During follow-up, there was a slight increase in hematocrit in both groups treated with dapagliflozin (without PAD and with PAD), while there was a slight decrease in hematocrit in the group of patients with PAD treated with other antidiabetic drugs. Statistically significant differences were observed in mean hematocrit values at the end of follow-up between dapagliflozin-treated patients without PAD and dapagliflozin-treated patients with PAD (0.447 vs. 0.429, *p* = 0.016) and between patients without PAD treated with dapagliflozin and patients with PAD treated with other antidiabetic drugs (0.447 vs. 0.412, *p* = 0.001).

Hemoglobin levels were significantly higher in the dapagliflozin-treated patients without PAD than in the dapagliflozin-treated patients with PAD and the group of patients with PAD treated with other antidiabetic drugs, both at baseline (145.944 g/L vs. 135.614 g/L *p* = 0.009 and 145.944 g/L vs. 140.038 g/L, *p* = 0.018) and at follow-up (149.917 g/L vs. 142.371 g/L, *p* = 0.001 and 149.917 g/L vs. 136.808 g/L, *p* = 0.001), although hemoglobin levels increased in both groups treated with dapagliflozin and decreased in the group with PAD treated with other antidiabetic drugs.

LDL cholesterol levels were significantly higher in dapagliflozin-treated patients without PAD than in dapagliflozin-treated patients with PAD at follow-up (2.178 mmol/L, SD = 0.933 vs. 1.817, SD = 0.939, *p* = 0.024). In addition, non-HDL cholesterol levels at follow-up were also significantly higher in the group of dapagliflozin-treated patients without PAD than in the dapagliflozin-treated patients with PAD (2.883 mmol/L, SD = 1.064 vs. 2.474 mmol/L, SD = 0.983, *p* = 0.020).

Fasting glucose levels at baseline were significantly higher in the dapagliflozin-treated group with PAD than in the dapagliflozin-treated group without PAD and the group of patients with PAD treated with other antidiabetic drugs (9.231 vs. 8.194 mmol/L, *p* = 0.005 and 9.231 vs. 8.064 mmol/L *p* = 0.040), although the HbA1c values did not differ between the groups at baseline or follow-up.

None of the observed variables affected the statistical association (significance) between the outcome (tissue oxygenation) and explanatory variables, which means that, if a significant association was observed in the core analysis, it remained significant until the end of follow-up, and vice versa. Furthermore, regression factors were not changed considerably.

The mean value of TcPO2 in the subcutaneous tissue of the foot was highest at baseline in the group without PAD treated with dapagliflozin (51.292 mm Hg), and it was significantly higher than the TcPO2 value in the group with PAD treated with dapagliflozin (46.200 mm Hg, *p* = 0.002) and significantly higher than the TcPO2 value in the group with PAD treated with other antidiabetic medications (43.404 mm Hg, *p* = 0.0002, *p* = 0.001). No statistically significant difference was found between the mean TcPO2 values between the group with PAD treated with dapagliflozin and the group with PAD treated with other antidiabetic drugs (Table 3).

At follow-up, there was a decrease in TcPO2 values in all groups, with the highest mean control TcPO2 value in the group without PAD treated with dapagliflozin of 47.83 mm Hg, which was significantly higher than the mean control TcPO2 value in the group with PAD treated with dapagliflozin (42.314 mm Hg, *p* = 0.002) and significantly higher than in the group with PAD treated with other antidiabetic drugs (41.923 mm Hg, *p* = 0.001). The observed differences in the mean values of the control TcPO2 were not statistically different between the group with PAD treated with dapagliflozin and the group with PAD treated with other antidiabetic drugs (*p* = 0.495) (Table 4).

At follow-up, the largest decrease in mean TcPO2 was observed in the group with PAD treated with dapagliflozin (3.88 mm Hg), followed by the group without PAD treated with dapagliflozin (1.76 mm Hg), and the smallest decrease was observed in the group with PAD treated with other antidiabetic drugs (1.48 mm Hg). No statistically significant differences were found in the decrease in mean TcPO2 between the groups (Table 5).

**Figure 1:**
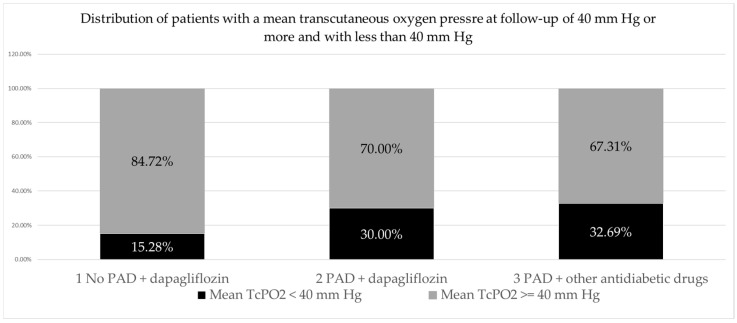
PAD, peripheral arterial disease.

When analyzing the distribution of patients (feet) with mean TcPO2 control values of less than 40 mm Hg and of 40 mm Hg or more by group, the largest proportion of patients (feet) had mean TcPO2 control values of ≥40 mm Hg (145 feet or 74.74% of the total number), while a smaller proportion of patients (feet) had mean TcPO2 control values < 40 mm Hg (49 feet or 25.25% of the total number). A slightly higher proportion of patients with mean control TcPO2 less than 40 mm Hg was found in the group with PAD treated with other antidiabetic drugs (17 feet; 32.69%) than in the group with PAD treated with dapagliflozin (21 feet, 30%), and the lowest proportion was found in the group without PAD treated with dapagliflozin (11 feet, 15.28%). The observed differences in the frequency of patients with TcPO2 < 40 mm Hg differed significantly between the three patient groups (*p* = 0.046), but not significantly between the two groups with PAD (2 vs. 3 *p* = 0.751). (Table 6, Figure 1).

Among patients with a control transcutaneous oxygen pressure of less than 40 mm Hg, the highest control TcPO2 was observed in the group without PAD treated with dapagliflozin (31.36 mm Hg, SD 7.47) than in the group with PAD treated with other antidiabetic drugs (28.70 mm Hg SD 9.10), and the lowest control TcPO2 level was in the group with PAD treated with dapagliflozin (27.71 mm Hg, SD 11.87). There was no significant difference in the control TcPO2 between groups (Table 7).

**Figure 2:**
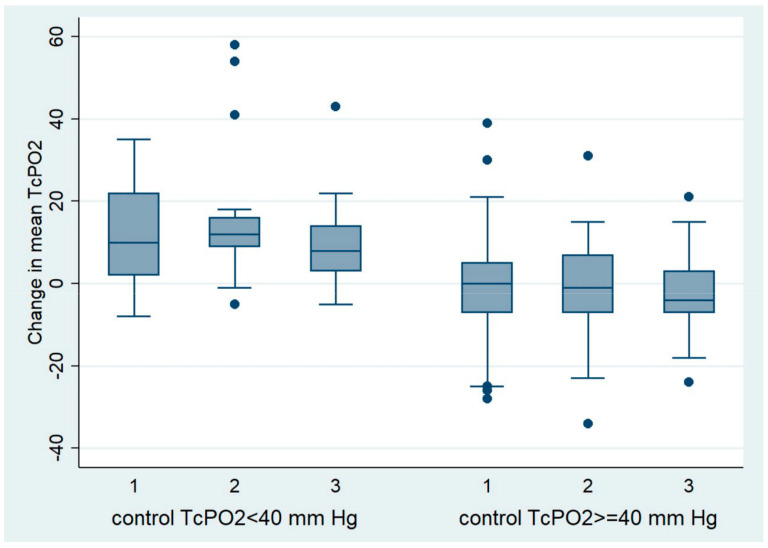
Change in mean TcPO2 at follow-up in patients with a control TcPO2 < 40 mm Hg and ≥40 mm Hg.

Among patients with a control TcPO2 of less than 40 mm Hg, the mean TcPO2 decreased from the beginning of the study to the control in all groups. The largest decrease in mean TcPO2 was observed in the group of patients with PAD treated with dapagliflozin (15.80 mm Hg) than in the group of patients without PAD treated with dapagliflozin (12.90 mm Hg), and the smallest decrease in TcPO2 was observed in the group of patients with PAD treated with other antidiabetic drugs (9.35 mm Hg). No significant differences in the degree of TcPO2 reduction were found between the observed groups (Table 8, Figure 2).

Among patients who had a control TcPO2 of 40 mm Hg or higher, mean TcPO2 during follow-up increased slightly in all groups; in the group without PAD treated with dapagliflozin, by 0.24 mm Hg; in the group with PAD treated with dapagliflozin, by 1.22 mm Hg; and in the group with PAD treated with other antidiabetic drugs, by 2.34 mm Hg. No significant differences in the degree of TcPO2 elevation were found between the observed groups (Table 9, Figure 2).

A certain number of ABI index measurements failed because the device was unable to measure the ABI value due to the low systolic pressure at the ankle level. Since the number of failed measurements was significant in the group with PAD treated with dapagliflozin (45.71%) and in the group with PAD treated with other antidiabetic drugs (53.85%), we did not include these groups in the ABI analysis. In the group of patients without PAD treated with dapagliflozin, there were 12.50% unsuccessful measurements, which is, in our opinion, an insignificant and acceptable proportion, and these measurements were included in the analysis. According to the guidelines of the Society for Vascular Surgery and the American Venous Forum, a change in ABI of 0.15 is required to be considered clinically relevant, or more than 0.10 if accompanied by a change in clinical status. In the group of patients without PAD who were treated with dapagliflozin (a total of 63 legs were analyzed), there was a minimal and non-significant increase in ABI value after follow-up compared to baseline (0.04609375, SD 0.3567072; i.e., from 1.202, SD 0.136 at baseline to 1.212, SD 0.087 at follow-up) [23].

In summary, the mean value of TcPO2 in the subcutaneous tissue of the foot was highest in the group without PAD (group 1) both at the beginning and at the end of the follow-up period, with a significant difference compared to both groups with PAD, while the TcPO2 finding in the foot was not significantly different between the group with PAD treated with dapagliflozin (group 2) and the group with PAD treated with other antidiabetic drugs (group 3), either at baseline or at the end of the follow-up period (Table 3 and Table 4). During the follow-up period, there was a slight decrease in TcPO2 values in all groups (1.76, 3.88, 1.48 mm Hg), with the decrease in TcPO2 values being more pronounced in the group with PAD treated with dapagliflozin, although it did not differ significantly between the groups (1−2 *p* = 0.130; 1–3 *p* = 0.414; 2–3 *p* = 0.106). In the group of patients in whom TcPO2 decreased below 40 mm Hg after follow-up, which we consider a pathologic finding reflecting hypoxia of the feet, the lowest TcPO2 was in the PAD group treated with dapagliflozin (group 2) (31.36, 27.71, 28.70 mm Hg) but with no significant difference compared to the other groups (1–2 *p* = 0.224; 1–3 *p* = 0.164; 2–3 *p* = 0.384). In the group of patients with pathologic TcPO2 control findings (<40 mm Hg), the extent of TcPO2 reduction in the feet was much more pronounced in all groups and most pronounced in the PAD group treated with dapagliflozin (12.9, 15.80, 9.35 mm Hg), but again with no significant difference between groups (1–2 *p* = 0.224; 1–3 *p* = 0.164; 2–3 *p* = 0.384). When we analyzed the proportion of patients with pathological TcPO2 control findings (TcPO2 < 40 mm Hg) by group, we found no significant differences between the groups with PAD (32.69% vs. 30%, *p* = 0.751).

## 3. Discussion

In patients with diabetes, PAD develops early, progresses rapidly, and is often asymptomatic. The atherosclerotic process is more pronounced in the distal arteries of the legs (tibial artery, dorsalis pedis artery) with impaired microcirculation of the feet compared to patients with PAD without diabetes [2,24]. Together with diabetic neuropathy, this could at least partially explain the significantly higher incidence of non-traumatic leg amputations in patients with PAD and diabetes compared to patients with PAD without diabetes.

SGLT-2 inhibitors are now considered one of the most important antidiabetic agents in T2DM and are particularly recommended for the treatment of patients with diabetes with cardiovascular and renal comorbidities or risks for these conditions due to their proven cardio–renal protective effect, regardless of the degree of diabetes control [24]. Due to the significant and twofold increased risk of amputations in patients treated with the SGLT2 inhibitor canagliflozin compared with placebo, as shown in the CANVAS study, and considering the potential class effect, caution is recommended when introducing SGLT2 inhibitors into the treatment of patients at increased risk of lower limb amputations, which includes patients with PAD [17,25,26]. This puts the clinician in an unenviable position where, guided by the Latin maxim “Primum non nocere” (First do no harm), they often decide not to use an SGLT2 inhibitor in patients with diabetes with PAD and/or diabetic foot. Considering this context, this study aimed to determine whether therapy with the SGLT2 inhibitor dapagliflozin leads to changes in peripheral vascular oxygenation, i.e., feet microcirculation, in patients with diabetes without PAD compared to patients with diabetes with PAD and compared to patients with diabetes with PAD treated with other antidiabetic agents, with the exception of thiazolidinediones, GLP-1 receptor agonists, and the combination of GLP-1 and GIP receptor agonists. We also wanted to see the potential connection between metabolic parameters and possible changes in foot microcirculation.

We used TcPO2 to measure oxygen pressure on the skin surface of the foot to assess peripheral vascular oxygenation and obtain information on microcirculatory function. The TcPO2 is used in clinical practice to quantify the severity of peripheral arterial disease, determine the optimal level of amputation, assess revascularization procedures, select candidates for hyperbaric oxygen therapy, and predict its efficacy. TcPO2 is a better predictor for ulcer healing than the toe-brachial index in patients with diabetes with chronic foot ulcers [27]. According to the consensus of professional societies, TcPO2 values below 40 mm Hg represent hypoxia of the skin and subcutaneous tissues of the foot, which slows and prevents wounds or residual limbs from healing, while values of 40 (50) mm Hg or higher are considered normal findings [10,11,12,13,28]. In patients with TcPO2 values below 40 mm Hg, it is recommended to measure the TcPO2 additionally after elevating the legs. A drop in TcPO2 values of 10 mm Hg or more is considered clinically significant in terms of increased risk of a non-healing wound or amputation [29].

There are numerous positive effects of SGLT-2 inhibitors on microcirculation. The strongest effect is their nephroprotective effect, with a proven relative risk reduction of 39% in composite renal outcomes (≥50% sustained decrease in eGFR, ESKD, renal, or CV death) [19]. The beneficial effects of dapagliflozin on the microvasculature in terms of reducing retinal capillary hyperperfusion and minimizing arteriole remodeling are evident after only 6 weeks of therapy with dapagliflozin compared to placebo [30]. Another study found that treatment with dapagliflozin improves endothelial function after 12 weeks of treatment compared to glibenclamide [31]. Compared to placebo, dapagliflozin as an add-on therapy to metformin for 16 weeks improved endothelial function in patients with inadequately controlled early-stage T2DM [32]. Even an acute treatment with dapagliflozin for two days significantly improves systemic endothelial function and arterial stiffness independently of blood pressure changes, suggesting a direct beneficial effect on the vasculature [33]. In patients with T2DM with underlying ischemic heart disease who were receiving metformin and insulin therapy, endothelial function in the group of patients with T2DM with an HbA1c > 7.0% at baseline was improved by additional therapy with dapagliflozin for 12 weeks compared to placebo [34]. As far as we know, the effects of SGLT2 inhibitor therapy on microcirculation in the feet have not yet been investigated using transcutaneous oximetry. As expected, TcPO2 at baseline showed a significantly better TcPO2 value in the group without PAD compared to the two groups with PAD (*p* = 0.002, *p* = 0.001). After an average follow-up period of 119.35 days, we observed a decrease in TcPO2 findings, i.e., a slight worsening of the microcirculation in all three patient groups. In the group with PAD treated with dapagliflozin, the decrease was more pronounced than in the group without PAD treated with dapagliflozin (3.88 mm Hg; 1.76 mm Hg), while the decrease in TcPO2 was the least pronounced in the group with PAD treated with other antidiabetic drugs (1.48 mm Hg).

Although we found no statistically significant difference in the magnitude of the decrease in TcPO2 levels between the groups, the question arises as to why there was no improvement in microcirculation in the dapagliflozin-treated groups, considering that numerous studies indicate a positive effect of dapagliflozin on microcirculation very soon after starting the medication, i.e., in a period of 42–112 days [30,31,32]. Simultaneously with the slight decrease in TcPO2 findings in the dapagliflozin-treated group without PAD, we noted a slight and insignificant improvement in ABI values, which neutralizes the effect of macrocirculation on the decrease in TcPO2 values. Unfortunately, we could not establish the aforementioned correlation between ABI and TcPO2 change in the groups with PAD due to technical limitations. A significant decrease in BMI and an increase in hemoglobin and hematocrit in the dapagliflozin-treated groups compared with the PAD group treated with other antidiabetic drugs could only have a positive effect on microcirculation. Slightly elevated levels of LDL and non-HDL cholesterol above target in all groups despite prescribed lipid-lowering therapy are the result of the small number of patients who did not take regular lipid-lowering therapy, and the significantly higher level of LDL and non-HDL cholesterol in the group without PAD treated with dapagliflozin compared to the groups with PAD is the result of less stringent lipid-lowering therapy according to their moderate to high degree of cardiovascular risk compared to the groups with PAD. However, their significant effect on the TcPO2 control values was excluded using stepwise regression to check for possible confounding variables. In the same way, possible confounding by other parameters that were statistically different between groups was also excluded (Table 1 and Table 2) [35,36,37].

When analyzing the distribution of patients (feet) with mean TcPO2 control values of less than 40 mm and 40 mm Hg or more by group, the largest proportion had mean TcPO2 control values of 40 mm Hg or more (145 feet or 74.74% of the total number), while a smaller proportion had mean TcPO2 control values of less than 40 mm Hg (49 feet or 25.25% of the total number). The observed differences in the frequency of patients with TcPO2 < 40 mm Hg differed significantly between the three patient groups (*p* = 0.046) but not significantly between the two groups with PAD (2 vs. 3 *p* = 0.751).

As mentioned previously, in patients with a TcPO2 of less than 40 mm Hg, an additional decrease in TcPO2 after elevation of the leg of 10 mm Hg or more is considered clinically significant in terms of an increased risk of a non-healing wound or amputation. When we separately analyzed patients with a pathological TcPO2 control value indicating hypoxia of the foot (TcPO2 < 40 mm Hg), the degree of decrease in TcPO2 was even more pronounced. There was a clinically significant decrease in control TcPO2 in the dapagliflozin-treated groups (15.80 mm Hg in the group with PAD and 12.9 mm Hg in the group without PAD), whereas in the group with PAD treated with other antidiabetic medications, this decrease was below clinical significance and amounted to 9.35 mm Hg (Table 7 and Table 8) [29]. Despite the clinically significant differences mentioned above, no significant differences between the groups were found in the statistical analysis. When we separately analyze patients whose control TcPO2 is normal (≥40 mm Hg), we find that there was a slight increase in TcPO2 values in all groups, i.e., an improvement in microcirculation, although the increase was insignificantly lower in the groups treated with dapagliflozin (without PAD 0.24 mm Hg, PAD 1.22 mm Hg) compared to the group with PAD treated with other antidiabetic drugs (2.34 mm Hg).

It can be further investigated with a larger number of patients whether and according to which metabolic, anamnestic, and therapeutic parameters the groups of patients with pathological and normal control TcPO2 values differ or whether there are additional factors that can be associated with such different dynamics of TcPO2 findings; i.e., the opposite effects on the microcirculation in all three groups of patients (Figure 1). For example, one possible explanation could be the nature of the other antidiabetic medications used to treat some of the patients in all three groups, such as DPP4 inhibitors, which have shown an antiatherosclerotic and vasculoprotective mechanism in clinical and experimental studies, or metformin, which has been shown to improve muscle microvascular insulin sensitivity in insulin-resistant humans and retinal capillary perfusion in diabetic mice [38,39,40].

In summary, patients with control TcPO2 findings suggestive of hypoxia (TcPO2 < 40 mm Hg) who were treated with dapagliflozin had a decrease in mean TcPO2 of 10 mm Hg or more, which is considered clinically significant. However, the aforementioned decrease in TcPO2 was not statistically significantly different from the decrease in TcPO2 in the group with PAD treated with other diabetic medications (*p* = 0.226, *p* = 0.094).

The present study has several potential limitations. First, this was a single hospital-based study with a limited number of study participants who most probably are not representative of the population with PAD. Therefore, the data must be confirmed in prospective studies with more patients. Second, the short follow-up time is an obvious limitation. Third, since our study only included patients from a white European population, there was no racial/ethnic diversity. Fourth, there was only one control measurement of the TcPO2 findings, which could be a limitation, especially if the changes in the TcPO2 findings are small. From the authors’ point of view, the main limitation of the presented research is the relatively small sample size, which can result in low statistical power, making it difficult to detect meaningful effects or relationships, large variability, and outliers in some measurements.

## 4. Materials and Methods 

### 4.1. Study Design and Subjects

This was a single-center prospective randomized pilot study conducted at the Polyclinic of the Vuk Vrhovac University Clinic for Diabetes, Endocrinology and Metabolic Diseases. This pilot study is part of a larger study that will last at least one year and include a larger number of patients. During this time, it is planned to repeat the TcPO2 measurements at 3- to 6-month intervals to monitor the dynamics of possible changes in the microcirculation of the feet over time and after therapeutic interventions.

This study initially included 97 T2DM patients who had previously been treated with standard antidiabetic therapy (metformin and/or DPP4 inhibitors and/or sulfonylureas and/or insulin), of whom 61 patients had PAD and 36 patients had no PAD. The patients with PAD were mostly recruited from the Department and Clinic of Vascular Surgery at the Merkur University Hospital and invited by telephone to the diabetology outpatient clinic for examination. A significantly smaller number of patients were discovered during examinations in the diabetology outpatient clinic, where they were referred by their GP for diabetes treatment. Patients without PAD were referred by their GP to the diabetology outpatient clinic for diabetes treatment and to investigate chronic diabetic complications.

**Figure 3:**
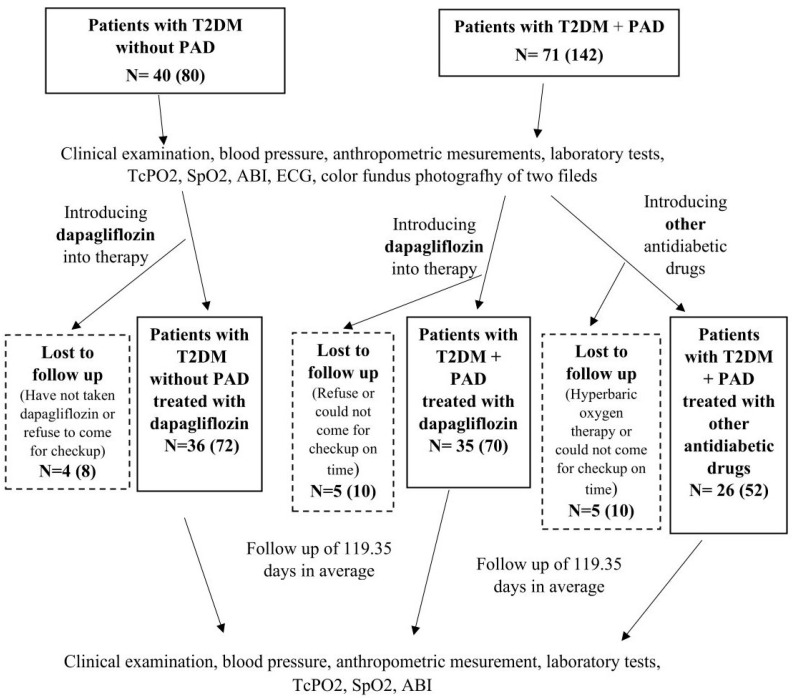
Study design diagram.

The inclusion criteria were patients aged 40–85 years with T2DM with or without PAD. Acute and severely ill patients, patients with T2DM with or without PAD treated with thiazolidinediones, GLP-1 receptor agonists, or a combination of GLP-1 and GIP receptor agonists, patients with acute lower limb ischemia, patients who had undergone peripheral revascularization or angiography within the last 4 weeks, patients with advanced chronic kidney disease with an eGFR < 15 mL/min/1.73 m^2^ and/or chronic dialysis therapy, patients with active malignant disease, and patients with scleroderma were not included in this study. Patients who underwent additional peripheral revascularization and/or hyperbaric oxygen therapy during the study (n = 1) or who were diagnosed with malignant disease and had started antineoplastic treatment, as well as patients who had started treatment with thiazolidinedione, GLP-1 receptor agonists, or a combination of GLP-1 and GIP receptor agonists, were excluded from the study. Patients with PAD were randomized into two groups according to the order of arrival at the diabetes outpatient clinic. In the first 22 patients with PAD, dapagliflozin was added to the therapy; in the next 22 patients with PAD, other antidiabetic drugs were added to the therapy, and then the procedure was repeated (Figure 3). Dapagliflozin was added to therapy in all patients without PAD.

At the beginning of the study, before the therapeutic intervention, TcPO2, ankle-brachial index (ABI), peripheral oxygen saturation (SpO2), color fundus photography of two fields (macula-centered, optic disc-centered) of both eyes with a standard VISUCAM Zeiss camera, blood pressure, anthropometric parameters, and laboratory tests were measured in all patients, which were repeated after a follow-up period. In the data analysis, TcPO2 was evaluated separately for each leg; i.e., a total of 194 legs were analyzed, 122 in the group of patients with PAD and 72 in the group of patients without PAD (Figure 3).

In clinical practice, a single TcPO2 measurement on the foot is a sufficient finding to provide an indication for hyperbaric oxygen therapy. In recent studies, microcirculation at the foot was assessed by a single TcPO2 measurement [28,41]. Therefore, we believe that, for a pilot study and considering the number of legs included in the analysis (194), one TcPO2 measurement is sufficient for the assessment of microcirculation at the foot.

ABI measurements were performed on all 194 legs. A certain number of ABI index measurements failed because the device could not measure the ABI value due to the low systolic pressure at ankle level. Therefore, the results of the successful measurements on a total of 63 legs were analyzed.

All patients in the group with PAD were at very high cardiovascular risk, including patients in the group without PAD who had documented cerebrovascular and/or ischemic heart disease. Other patients in the group without PAD had moderate or high cardiovascular risk. Lipid-lowering therapy (statins with or without ezetimibe) was recommended to all patients according to estimated cardiovascular risk, and therapy with ACE inhibitors or ARBs according to the 2024 ADA guidelines and antiplatelet and/or anticoagulant therapy were recommended for all patients with PAD and/or cerebrovascular and/or cardiovascular disease.

Abbreviations used in the text are as follows: T2DM, diabetes mellitus type 2; PAD, peripheral arterial disease; TcPO2, transcutaneous oxygen pressure; SpO2, peripheral oxygen saturation; ABI, Ankle Brachial index; and ECG, electrocardiogram.

### 4.2. Data Collection

When taking the medical history, information on chronic diseases, smoking status, and medication intake was obtained. All data and results were stored via the hospital information system. Those who currently smoked or who had smoked at least 100 cigarettes in the past were defined as smokers. Hypertension was defined as a systolic blood pressure (SBP) ≥ 140 mm Hg and/or diastolic blood pressure (DBP) ≥ 90 mm Hg or antihypertensive treatment. Body mass index (BMI) was calculated by dividing weight in kilograms and height in meters squared. Waist circumference (WC) and hip circumference were measured with a tailor’s tape measure at standard sites on bare skin to calculate the waist-to-hip ratio (WHR).

Complete blood count (CBC), white blood cell differential, prothrombin time (PT), prothrombin time—international normalized ratio (PT-INR), activated partial thromboplastin time (APTT), fibrinogen level test, D-dimer test, potassium, sodium, chloride, ionized calcium, total calcium, inorganic phosphate, iron, total iron binding capacity, unsaturated iron binding capacity, urea, creatinine, urate, total bilirubin, aspartate aminotransferase, alanine aminotransferase, gamma-glutamyl transferase, alkaline phosphatase, total cholesterol, high-density lipoprotein (HDL), low-density lipoprotein (LDL), very low-density lipoprotein (VLDL), triglycerides, non-HDL cholesterol, lipoprotein (a), proteins, albumin, C-reactive protein, proteins and creatinine in a single urine sample, protein/creatinine ratio, albumin/creatinine ratio, and urine biochemistry and sediment were measured on the day of hospital admission. All samples were collected after a 12-h fasting period.

Glycated hemoglobin (HbA1c) was measured using an automated turbidimetric inhibition immunoassay (HbA1c Gen 3, Cobas Integra 400 Plus, Roche Diagnostic, Basel, Switzerland) and expressed as a percentage according to the National Glycohemoglobin Standardization Program (NGSP).

### 4.3. Diagnosis of Peripheral Arterial Disease

Most patients with PAD were diagnosed by multislice spiral computed tomography (MSCT) and/or digital subtraction angiography, and most had previously undergone peripheral revascularization procedures. In a small number of patients, PAD was diagnosed by ultrasound Doppler, where the absence of a normal triphasic appearance of the signals, i.e., the presence of a biphasic and/or monophasic appearance of the signals in one of the lower limb arteries, was considered as PAD. 

### 4.4. Microcirculation Assessment—Transcutaneous Oxygen Pressure Measurement

The sensor/electrode of the TCM5 series transcutaneous monitor (Radiometer, Denmark) was placed on the skin, where it heated the underlying tissue to produce local hyperemia (causing the artery to dilate), intensifying blood flow and increasing oxygen levels. The electrode was heated to 45 °C and delivered a temperature of about 43 °C to the skin, which improved the oxygenation of the capillary blood. The sensor received an electric current that corresponded to the oxygen concentration in the capillary blood. Depending on the pressure, the oxygen diffused from the capillary blood through the vascular epidermis to the electrode placed on the skin surface. After application, the sensor required a warm-up phase of 10 to 15 min and had to be calibrated every four to eight hours. An on-site measurement took an average of 35 min per leg. The sensor was placed on a homogeneous capillary bed without hair, skin defects, ulcers, or protruding veins. The electrode was not placed over bone, previous surgical sites, scar tissue, or severe edema, as this would have led to unreliable results. Patients were advised not to smoke or consume caffeine for at least one hour before the test. Patients were placed in the supine position during monitoring.

### 4.5. Ankle Brachial Index

This index is calculated by comparing the highest measured systolic pressure in the ankle area with the brachial systolic pressure, and their ratio gives the ABI. This index provides us with relative information about the severity of the peripheral arterial occlusive disease. Before starting the examination, the patient rests for at least 15 min in a supine position with the limbs parallel to the body. The MESI mTABLET device with blood pressure diagnostic module model/type TBPMD, serial number 1211-MDGO, manufactured in Ljubljana, Slovenia, consisting of four cuffs applied simultaneously to both upper arms and both ankles, was used to measure the ABI.

### 4.6. Fundus Photography

Most patients underwent fundus photography with a standard 45° VISUCAM Zeiss fundus camera (Carl Zeiss Meditec AG, Jena, Germany). Patients who underwent ophthalmologic monitoring and treatment at other institutions did not undergo fundus photography, but their medical records from another institution were reviewed. For all patients who underwent fundus photography, the images were analyzed by an ophthalmologist and a posterior segment subspecialist, and a report was written.

### 4.7. Statistical Analysis

We processed the numerical indicators statistically using the statistical program Stata 13.1 (Stata Corp., College Station, TX, USA). We checked the normality of the distribution of indicators expressed in continuous values using the Shapiro–Wilk test. We compared the values of each indicator between three groups of patients: (1). patients without PAD treated with dapagliflozin; (2). patients with PAD treated with dapagliflozin; and (3). patients with PAD treated with other antidiabetic drugs. In the statistical analysis of tissue oximetry, we considered each limb individually. We checked the statistical significance of differences in each indicator expressed in continuous values between patient groups using analysis of variance for indicators that met the criteria of normal distribution (Shapiro–Wilk test *p* > 0.05), and for indicators that did not meet the criteria of normal distribution (Shapiro–Wilk test *p* < 0.05), we used the non-parametric Kruskal–Wallis test. Tukey’s post-hoc test and Dunn’s test were used to compare groups after parametric and non-parametric statistical analysis. We tested the significance of differences in indicators expressed in binary values (yes/no) using the chi-square test and Fisher’s exact test. Stepwise regression was performed to check possible confounding variables. Before regression analysis, variables were log transformed in order to normalize distribution.

## 5. Conclusions

Based on the available data, dapagliflozin appears to have an effect on tissue oxygenation in T2DM with PAD. The results show differences in TcPO2 control values between patients treated with dapagliflozin and those treated with other blood-glucose-lowering drugs. To better understand the specific effects of dapagliflozin on tissue oxygenation in the feet of T2DM patients with PAD, further studies and analyses are needed, in particular by extending the follow-up period and repeating the TcPO2 examinations and ideally by including a larger number of patients.

## Figures and Tables

**Table 1:** The intergroup comparisons of all patients at the beginning of the follow-up.

Baseline Characteristics of All Patients (*n* = 97)					
Without Peripheral Artery		Disease With Peripheral Artery Disease			
*n* = 36		*n* = 61			
Dapagliflozin		Dapagliflozin *n* = 35	Other Antidiabetics *n* = 26	*p* Value Group 2 vs. 3	*p* Value All Groups
Age, median	67.5	70	71.5	*p* = 0.780	*p* = 0.817
Female sex (*n*)	10	8	7	*p* = 0.770	*p* = 0.877
Body mass index, mean (SD)	30.387 (5.717)	28.999 (7.453)	26.571 (3.742)	*p* = 0.0075	*p* = 0.012
History hypertension, (*n*) (%)	31 (31.96)	34 (35.05)	23 (23.71)	*p* = 0.303	*p* = 0.290
History hyperlipidemia, *n* (%)	30 (30.93)	33 (34.02)	17 (17.53)	*p* = 0.006	*p* = 0.015
History diabetic polyneuropathy, *n* (%)	16 (16.46)	29 (29.90)	22 (22.68)	*p* = 1	*p* = 0.001
History diabetic retinopathy, *n* (%)	7 (7.22)	14 (14.43)	8 (8.29)	*p* = 0.459	*p* = 0.166
Smoker, *n* (%)	16 (16.49)	26 (26.80)	20 (20.62)	*p* = 0.813	*p* = 0.009
Hemoglobin A1c mean (SD),	7.350 (1.289)	7.314 (1.506)	7.062 (1.452)	*p* = 0.320	*p* = 0.200
Duration of diabetes mellitus, (y) mean (SD)	8.694 (7.920)	16.829 (9.724)	12.462 (7.393)	*p* = 0.248	*p* = 0.216
Estimated GFR (CKD-EPI) mL/min/1.73 m^2^, *n* (%)	79.028 (19.191)	73.400 (18.753)	73.692 (22.922)	*p* = 0.455	*p* = 0.476
History of ischemic heart disease, *n* (%)	3 (3.09)	14 (14.43)	6 (6.19)	*p* = 0.164	*p* = 0.006
History of myocardial infarction, *n* (%)	1 (1.03)	11 (11.34)	4 (4.12)	*p* = 0.230	*p* = 0.004
History of cerebrovascular disease, *n* (%)	4 (4.12)	17 (17.53)	13 (13.4)	*p* = 0.912	*p* = 0.001
History of cerebral infarction, *n* (%)	3 (3.09)	7 (7.22)	5 (5.15)	*p* = 0.940	*p* = 0.314
Antiaggregation and/or anticoagulation therapy, *n* (%)	9 (9.28)	32 (32.99)	21 (21.65)	*p* = 0.268	*p* = 0.001
Lipid-lowering therapy, *n* (%)	27 (27.84)	27 (27.84)	13 (13.40)	*p* = 0.027	*p* = 0.048
ACE inhibitor or ARB therapy, *n* (%)	27 (27.84)	31 (31.96)	19 (19.59)	*p* = 0.179	*p* = 0.242
**Peripheral artery disease history**					
Previous peripheral revascularization (endovascular or surgical), *n* (%)		24 (24.74)	17 (17.52)	*p* = 0.796	
Previous amputation, *n* (%)		9 (9.28)	1 (1.03)	*p* = 0.034	
**Fontaine Classification at randomization**					
Stage I: Asymptomatic, *n* (%)		7 (11.48)	1 (1.64)	*p* = 0.081	
Stage II: Claudication, *n* (%)		21 (34.43)	22 (36.07)		
Stage III: Ischemia rest pain, *n* (%)		0	0		
Stage IV: Ulceration or gangrene, *n* (%)		7 (11.48)	3 (4.92)	*p* > 0.05	
SINBAD score		2.42	3		
**Ankle Brachial index category/leg**					
<0.9, *n* (%)	0	14 (11.20)	9 (7.20)	*p* = 0.958	*p* = 0.001
≥0.9, *n* (%)	63	24 (19.20)	15 (12.0)		
**Transcutaneous oxygen pressure** **category/leg, mean, mm Hg**	51.292 (11.150)	46.200 (10.774)	43.404 (9.828)	*p* = 0.082	*p* = 0.002

**Table 2:** The intergroup comparisons of patients with and without peripheral arterial disease at the beginning and at follow-up.

		No PAD + DAPAGLIFLOZIN (1)		PAD + DAPAGLIFLOZIN (2)		PAD + Other Antidiabetic Drugs (3)		*p* Value		
								1–2	1–3	2–3
		mean	sd	mean	sd	mean	sd			
BMI (kg/m^2^)	baseline	30.387	5.717	28.999	7.453	26.571	3.742	0.354	0.002	0.0075
	follow up	29.936	5.508	28.604	7.671	26.573	3.603	0.326	0.010	0.029
HbA1c (%)	baseline	7.350	1.289	7.314	1.506	7.062	1.452	0.362	0.165	0.320
	follow up	6.747	0.586	6.786	0.938	6.815	1.215	0.286	0.246	0.430
Glucose fasting (mmol/L)	baseline	8.194	1.779	9.231	2.603	8.064	2.073	0.005	0.380	0.040
	follow up	8.036	1.620	7.777	1.478	8.588	2.811	0.276	0.473	0.318
Hematocrit (L/L)	baseline	0.431	0.003	0.419	0.035	0.424	0.037	0.077	0.414	0.401
	follow up	0.447	0.032	0.429	0.033	0.412	0.043	0.016	0.001	0.059
Hemoglobin (g/L)	baseline	145.944	13.852	135.614	27.125	140.038	12.032	0.009	0.018	0.471
	follow up	149.917	13.693	142.371	11.971	136.808	14.006	0.001	0.001	0.081
LDL cholesterol (mmol/L)	baseline	2.489	1.146	2.489	1.162	2.531	0.995	0.452	0.462	0.395
	follow up	2.178	0.933	1.817	0.936	1.900	0.940	0.024	0.061	0.392
Total cholesterol (mmol/L)	baseline	4.447	1.325	4.543	1.367	4.492	1.322	0.425	0.437	0.494
	follow up	4.175	1.250	3.760	1.041	4.504	3.749	0.054	0.108	0.403
Non-HDL cholesterol (mmol/L)	baseline	3.208	1.248	3.251	1.337	3.196	1.185	0.481	0.475	0.493
	follow up	2.883	1.064	2.474	0.983	2.504	0.967	0.020	0.05	0.406
HDL cholesterol (mmol/L)	baseline	1.239	0.289	1.291	0.342	1.298	0.409	0.237	0.396	0.346
	follow up	1.292	0.285	1.337	0.469	1.242	0.412	0.466	0.120	0.138
Triglycerides (mmol/L)	baseline	1.602	0.692	1.943	2.364	1.455	0.740	0.419	0.106	0.077
	follow up	1.567	0.770	1.440	0.591	1.315	0.683	0.31	0.031	0.081
C-reactive protein (mg/L)	baseline	2.923	3.113	5.397	9.204	3.212	4.160	0.198	0.36	0.335
	follow up	3.044	3.943	3.674	4.648	3.050	3.717	0.488	0.495	0.494
Creatinine (umol/L)	baseline	85.028	20.740	89.711	36.187	89.231	38.998	0.292	0.438	0.372
	follow up	85.417	21.089	100.829	43.377	91.500	38.801	0.055	0.431	0.097

PAD, peripheral arterial disease.

**Table 3:** Transcutaneous oxygen pressure at baseline.

GROUP	Mean/mm Hg	SD	*n*
1 No PAD + dapagliflozin	51.292	11.150	72
2 PAD + dapagliflozin	46.200	10.774	70
3 PAD + other antidiabetic drugs	43.404	9.828	52
Total	46.96	10.58	194

PAD, peripheral arterial disease; differences between groups: 1–2 *p* = 0.002; 1–3 *p* = 0.001; 2–3 *p* = 0.082.

**Table 4:** Transcutaneous oxygen pressure at follow-up.

GROUP	Mean/mm Hg	SD	*n*
1 No PAD + dapagliflozin	49.528	12.238	72
2 PAD + dapagliflozin	42.314	13.304	70
3 PAD + other antidiabetic drugs	41.923	11.424	52
Total	44.58	12.322	194

PAD, peripheral arterial disease; differences between groups 1–2 *p* = 0.002; 1–3 *p* = 0.001; 2–3 *p* = 0.495.

**Table 5:** Decrease in mean transcutaneous oxygen pressure at follow-up.

GROUP	Mean/mm Hg	SD	*n*
1 No PAD + dapagliflozin	1.76	12.93	72
2 PAD + dapagliflozin	3.88	15.13	70
3 PAD + other antidiabetic drugs	1.48	11.55	52
Total	2.4536082	13.41372	194

PAD, peripheral arterial disease; differences between groups: *p* = 0.379; 1–2 *p* = 0.130; 1–3 *p* = 0.414; 2–3 *p* = 0.106.

**Table 6:** Distribution of patients with a mean transcutaneous oxygen pressure at follow-up of 40 mm Hg or more and with less than 40 mm Hg.

GROUP	Mean TcPO2 < 40 mm Hg *n* (%)	Mean TcPO2 ≥ 40 Mm Hg *n* (%)	Total
1 No PAD + dapagliflozin	11 (15.28)	61 (84.75)	72
2 PAD + dapagliflozin	21 (30)	49 (70)	70
3 PAD + other antidiabetic drugs	17 (32.69)	35 (67.31)	52
Total	49	145	194

PAD, peripheral arterial disease; differences between groups *p* = 0.046; 2–3 *p* = 0.751.

**Table 7:** Transcutaneous oxygen pressure at follow-up in patients with a control transcutaneous oxygen pressure of less than 40 mm Hg.

GROUP	Mean	SD	*n*
1 No PAD + dapagliflozin	31.36	7.47	11
2 PAD + dapagliflozin	27.71	11.87	21
3 PAD + other antidiabetic drugs	28.70	9.10	17
Total	28.87	10.00	49

PAD, peripheral arterial disease; differences between groups: 1–2 *p* = 0.224; 1–3 *p* = 0.164; 2–3 *p* = 0.384.

**Table 8:** Decrease in mean transcutaneous oxygen pressure at follow-up in patients with a control transcutaneous oxygen pressure of less than 40 mm Hg.

GROUP	Mean	SD	*n*
1 No PAD + dapagliflozin	12.909091	14.046028	11
2 PAD + dapagliflozin	15.809524	16.064305	21
3 PAD + other antidiabetic drugs	9.3529412	11.999694	17
Total	12.918367	14.310306	49

PAD, peripheral arterial disease; differences between groups are not statistically significant *p* > 0.05; 1–2 *p* = 0.355; 1–3 *p* = 0.226; 2–3 *p* = 0.094.

**Table 9:** Change in mean transcutaneous oxygen pressure at follow-up in patients with a control transcutaneous oxygen pressure ≥40 mm Hg.

GROUP	Mean/mm Hg	SD	*n*
1 No PAD + dapagliflozin	−0.24590164	11.75677	61
2 PAD + dapagliflozin	−1.2244898	11.53304	49
3 PAD + other antidiabetic drugs	−2.3428571	9.298775	35
Total	−1.0827586	11.09210	145

PAD, peripheral arterial disease; differences between groups: 1–2 *p* = 0.451; 1–3 *p* = 0.128; 2–3 *p* = 0.162.

## Data Availability

The data presented in this study are available on request from the corresponding author.
